# Pharmacy professionals’ knowledge, attitudes, and behaviours regarding antibiotic use and antimicrobial resistance in Oman

**DOI:** 10.1080/20523211.2026.2737505

**Published:** 2026-09-30

**Authors:** Zainab Said Al-Hashimy, Barbara R. Conway, Marwa Al Qasmi, Huda Al Harthi, Rashid Al Harthi, Feras Darwish Elhajji, Mamoon A. Aldeyab

**Affiliations:** a Department of Clinical Pharmacy, Directorate of Pharmaceutical Care and Medical Stores, Khoula Hospital, Muscat, Oman; b Department of Pharmacy, School of Applied Sciences, University of Huddersfield, Huddersfield, UK; c Department of Inpatient and Outpatient Pharmacy, Directorate of Pharmaceutical Care and Medical Stores, Khoula Hospital, Muscat, Oman; d Faculty of Pharmacy, Applied Science Private University, Amman, Jordan; e Reading School of Pharmacy, University of Reading, Reading, UK

**Keywords:** Antimicrobial resistance, antimicrobial stewardship, attitudes, behaviours, knowledge, pharmacy professionals

## Abstract

**Background:**

This study evaluated pharmacy professionals’ knowledge, attitudes, and behaviours regarding antibiotic use and antimicrobial resistance (AMR) in Oman.

**Methods:**

A cross-sectional, questionnaire-based survey was conducted among 411 pharmacy professionals in Ministry of Health (MOH) institutions from January to June 2023. This questionnaire is based on instruments developed by the European Centre for Disease Prevention and Control and assesses knowledge, opportunities, behaviours, and awareness of national initiatives.

**Results:**

A total of 411 pharmacy professionals participated in the study. Overall, participants demonstrated knowledge of AMR, with an understanding of antibiotic effectiveness against viruses (89.3%) and inappropriate use (96.4%), but important gaps in knowledge of resistance transmission (44.8%) and antibiotic use for growth promotion in farm animals (31.4%). Higher median knowledge scores were observed among senior specialist pharmacists (p<0.001), while respondents aged 36–45 years and those with 16–20 years of practice achieved the highest proportions of full knowledge scores (p<0.001). Although 91% of respondents reported confidence in their understanding of AMR, specific knowledge gaps were identified: 56.2% were unsure whether antibiotic use for growth promotion in farm animals was legal in Oman, and 36% were unaware of or unsure about Oman’s National Action Plan. Most respondents reported having opportunities to provide patient counselling (82%) and access to guidelines (63%), while 59.9% reported frequent antibiotic dispensing in practice. Importantly, among the 217 respondents who reported that the information changed their views, 208 (95.9%) participants indicated that this knowledge influenced their dispensing practices.

**Conclusions:**

Pharmacy professionals in Oman demonstrated awareness and positive perceptions regarding antibiotic use and antimicrobial resistance, although gaps remained in awareness and understanding of One Health principles, national initiatives, and the translation of knowledge into dispensing practice. Strengthening targeted education, guideline access, and pharmacist involvement in antimicrobial stewardship may improve appropriate antibiotic use and support AMR control.

## 1. Introduction

Antimicrobial resistance (AMR) represents a critical challenge in the management of infectious diseases worldwide. The phenomenon undermines the clinical utility of antibiotics, antivirals, and antifungals, a process accelerated by the chronic overuse and misuse of these agents(O'Neill, 2014). AMR leads to increased hospital stays, more complex and expensive treatment, and a high rate of complications, which increase the morbidity, mortality, and healthcare costs (O'Neill, 2014, 2016). A framework was developed to guide researchers in designing One Health studies by integrating human, animal, and environmental health through multidisciplinary collaboration, careful study planning, and appropriate analytical methods (Lebov et al., 2017). In addition, the stronger integrated One Health governance, linking these systems, is essential to prevent future pandemics and overcome existing legal, political, and financial challenges in global health security (Elnaiem et al., 2023).

Antimicrobial stewardship (AMS) emphasises the importance of responsible antibiotic use and highlights strategies such as improving prescribing practices, better diagnostics, and pharmacist-led stewardship programmes (O'Neill, 2016). Pharmacist-led antimicrobial stewardship programmes improved adherence to antibiotic guidelines and reduced inappropriate antibiotic prescription, and decreased antibiotic utilisation, resulting in shortened hospital stays, lowered healthcare costs, and improved clinical outcomes (Otieno et al., 2022). In addition, pharmacist-led interventions significantly increased the appropriate antibiotic prescribing quality, adherence to the guidelines, accelerated appropriate treatment, and supported better clinical decision-making (Fay et al., 2019; Kooda et al., 2022).

Pharmacists are integral members of AMS teams and contribute to guideline development, antimicrobial therapy optimisation, staff education, and antimicrobial use monitoring (Garau & Bassetti, 2018; Parente & Morton, 2018).

Collaboration between physicians and pharmacists within antimicrobial stewardship (AMS) programmes can substantially enhance the quality of antibiotic prescribing and contribute to reducing antimicrobial resistance. This collaboration should begin with the development and implementation of formal, locally adapted prescribing protocols and guidelines, ensuring that both professions actively participate in establishing and adhering to shared standards of practice (Saha et al., 2020). Findings from previous studies indicate significant pharmacist knowledge deficiencies regarding antibiotic indications, the global impact of AMR, weak regulatory enforcement for dispensing antibiotics, inconsistent practices, and limited collaboration with prescribers, as well as public misconceptions (Hafez et al., 2024).

In Oman, the Ministry of Health (MOH) established a foundation for these efforts by introducing the National Action Plan on Antimicrobial Resistance in 2016, which explicitly adopts a One Health approach and highlights capacity building and education as key strategic priorities to improve stewardship (Ministry of Health & Ministry of Agriculture & Fisheries, 2016). Despite growing global attention to AMR and AMS, limited evidence is available regarding the knowledge, attitudes, and behaviours of pharmacy professionals in Oman. In addition, awareness and implementation of the One Health approach remain underexplored in the local context. Challenges such as limited AMR-related training opportunities, variability in stewardship practices, and inconsistent multidisciplinary collaboration may affect the effective implementation of AMS initiatives in Oman. Identifying these gaps is important to support targeted educational interventions, strengthen stewardship programmes, and inform national AMR control strategies.

This study aimed to assess pharmacy professionals’ knowledge, perceived capability, opportunity, motivation, and self-reported behaviours regarding antibiotic use and AMR in Oman using the COM-B framework.

## 2. Methods

### 2.1. Study design

The survey was distributed to pharmacy professionals at the MOH institutions in Oman from 15 January to 25 June 2023. The MOH is the government sector providing care across the country free of charge, comprising health care centres, polyclinics, specialised polyclinics, and secondary care hospitals, as well as regional and tertiary care hospitals. In addition to the public healthcare system, Oman has a well-established private sector that provides healthcare services. In Oman, the MOH pharmacy workforce is classified into three distinct professional categories based on educational qualification and scope of practice: Senior Specialist Pharmacists (Master’s degree holders), Pharmacists (holding an M.Pharm, D.Pharm, or Bachelor’s degree), and Assistant Pharmacists (Diploma in Pharmacy). All categories are responsible for reviewing and dispensing prescriptions; however, prescribing authority remains exclusively limited to physicians. Senior Specialist Pharmacists possess an expanded clinical role. Their responsibilities include conducting comprehensive patient chart reviews, participating in multidisciplinary ward rounds, and advising physicians on therapy selection, dosage optimisation, and patient monitoring. Throughout this study, the term ‘pharmacy professionals’ refers collectively to these three categories. The eligible sampling frame comprised all 1,949 pharmacy staff registered across MOH institutions, as per the 2021 Annual Health Report (which administratively aggregates 1,167 assistant pharmacists and 782 pharmacists and specialists). Oman’s healthcare system is predominantly public, structured into primary, secondary, and tertiary tiers, where medications are prescribed through the national electronic health record system (Al Shifa) and dispensed through internal institutional pharmacies. In contrast, community pharmacies operate exclusively within the private sector. Under national pharmaceutical regulations, systemic antibiotics are classified strictly as prescription-Only medicines; dispensing antibiotics without a prescription is prohibited by law and subject to regulatory enforcement and penalties by the MOH. The questionnaires were derived from instruments developed by the European Centre for Disease Prevention and Control (ECDC) (Ashiru-Oredope et al., 2021; European Centre for Disease Prevention and Control. Survey of healthcare workers’ knowledge, attitudes and behaviours on antibiotics, antibiotic use and antibiotic resistance in the EU/EEA, 2019). In this study, the survey was offered in both English and Arabic to suit participants’ language preferences. The Arabic version was created through forward translation by bilingual clinical professionals and subsequently reviewed and pilot-tested by a panel of bilingual experts to ensure accuracy and the preservation of meaning, serving as the expert validation process for the local context. Since the survey was adapted from a validated European Centre for Disease Prevention and Control (ECDC) tool and maintained its original structure, this expert panel review was sufficient to ensure item equivalence. Additionally, because English is the official language of pharmacy education and clinical practice in Oman, offering both the original English version and the verified Arabic translation minimised the risk of misinterpretation. The survey was delivered via the Qualtrics platform, where participants could choose their preferred language, and system controls were set to restrict each participant to a single submission. Distribution was carried out through the MOH email system and WhatsApp messages. Through Qualtrics Forms, participants were invited to take part in the survey and access additional study information.

Participants were informed that participation was voluntary and that responses would be anonymous and confidential. Informed consent was obtained through implied consent; by voluntarily completing and submitting the survey, participants indicated their consent to participate. The study received approval from both the Health Studies and Research Approval Committee in Muscat, Oman (Moh/DGPS/CSR/PRO/approved/137/2022) and the University of Huddersfield Research Integrity and Ethics Committee (SAS-SRIEC-21.12.22-1).

### 2.2. Survey tool

The survey consisted of 36 items, including an opening section with 6 demographic information, including age, gender, governorate, profession, workplace, and years of experience, followed by 30 items assessing knowledge, attitudes, and behaviours related to antibiotic use and antimicrobial resistance. Its design was guided by the COM-B behavioural change framework, which posits that behaviour is shaped by interaction of capability (pharmacy professionals’ knowledge and skills), opportunity (external factors such as resources and social support), and motivation (internal processes, including reflective and automatic influences that shape decision-making and actions). The second section assessed participants’ knowledge through true-or-false items: four questions addressed awareness of antibiotic use, inappropriate use, and potential adverse effects; three focused on antibiotic resistance; and one examined antibiotic use within the animal sector. Correct answers earned 1 point on a scale from 0 to 8.

Participants in the third section responded using a 5-point Likert scale, extending from strongly agree to strongly disagree, along with options for ‘not applicable’ or ‘I don’t understand’. The section contained two statements addressing food and environmental aspects of antibiotic resistance, three statements on self-perceived knowledge, three statements on opportunities influencing prudent antibiotic use and infection management, and two statements on motivational opportunities to promote positive behaviours for AMR prevention and control.

The fourth section included three items addressing opportunities to supply antibiotics or resources that support their prudent use. Participants were asked about the frequency of behaviours over the past week, with response options including once a day, more than once a day, once a week, more than once a week, never, rarely, ‘I do not remember’, and ‘not applicable’.

The fifth section explored the resources utilised in infection management. The sixth section investigated whether participants had received guidance on avoiding unnecessary antibiotic dispensing and whether this influenced their attitudes and practices. The seventh section discussed sources of information used to enhance antibiotic dispensing. The final section addressed awareness of initiatives and action plans within the country. Additionally, a final question was included regarding topics of interest to pharmacy professionals.

### 2.3. Statistical analysis

The Raosoft sample size calculator (http://www.raosoft.com/samplesize.html) was used to estimate the sample size, with parameters set to a 5% margin of error, a 95% confidence level, and a 50% response distribution. All registered pharmacy professionals (n = 1,949) were eligible to participate, and invitations were distributed without restriction to the full sampling frame. The minimum required sample size, calculated a priori using a sample size calculator, was 322 participants. A total of 411 completed responses were received, exceeding the required sample size, for an achieved response rate of 21.1% (411/1,949). Information related to the number of participants was obtained from the MOH register. The data were analysed using IBM SPSS Statistics software (version 28.0, Armonk, NY, USA). Respondents’ sociodemographic characteristics were summarised using frequencies and percentages. The impact of demographic factors on knowledge scores was described with medians and interquartile ranges and assessed using either the Wilcoxon rank-sum test or the Kruskal–Wallis test, depending on data distribution. For the knowledge assessment, each correct response was assigned a score of 1, and each incorrect response was scored 0. The total knowledge score was calculated from eight knowledge-related questions, with possible scores ranging from 0 to 8. Chi-square tests or Fisher’s exact tests were utilised to evaluate associations between demographic groups and the categorical proportions of specific item responses. To identify independent predictors of continuous knowledge scores and adjust for confounding variables, univariable and multivariable linear regression analyses were performed. Prior to running the regression models, standard assumptions, including normality of residuals and homoscedasticity, were verified using residual Q-Q plots and scatterplots to ensure model stability for the bounded score. A *P*-value of less than 0.05 is considered significant at a 95% confidence level.

## 3. Results

### 3.1. Key finding

#### 3.1.1. Demographic data

A total of 411 pharmacy professionals responded to the survey. About 73% of the respondents were female, and around 49% were aged 24–35. Muscat governorate had the highest proportion of participants at 46%, and 49% of the total sample were pharmacists. The majority of respondents (56%) worked in the hospital, and 27% had 11–15 years of experience (Table 1).

**Table 1.** Sociodemographic characteristics of Respondents (n = 411).

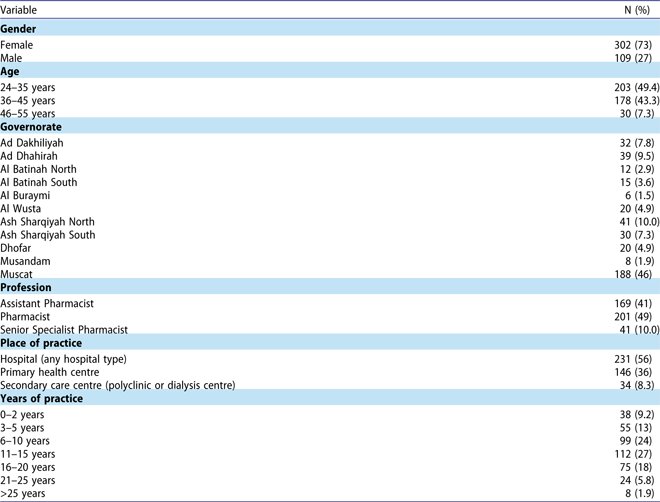

#### 3.1.2. Actual capability and perceived capability

Table 2 presents eight questions assessing the respondents’ actual knowledge. More than 96% answered correctly regarding the unnecessary use and side effects associated with antibiotic intake. However, 89.3% answered correctly about the effectiveness of antibiotics against viruses and 69.8% of them were able to answer regarding the effectiveness against colds. The respondents (66.2%) answered correctly regarding healthy people's ability to carry antibiotic resistance. However, only 44.8% of respondents answered the question about the spread of antibiotic resistance, and 74% answered correctly about the risk of antibiotic resistance while on treatment. Finally, 31.4% of the responders knew that the use of antibiotics as growth promoters in farm animals was illegal.

**Table 2.** Actual knowledge of Respondents (n = 411).

Key Knowledge Question	Correct Answer	True* n (%)	False* n (%)	Unsure* n (%)
Antibiotics are effective against viruses.	False	34 (8.3)	367 (89.3)	10 (2.4)
Antibiotics are effective against cold infections.	False	90 (21.9)	287 (69.8)	34 (8.3)
The unnecessary use of antibiotics makes them become ineffective.	True	396 (96.4)	10 (2.4)	5 (1.2)
Taking antibiotics has associated side effects or risks such as diarrhea, colitis, allergies.	True	395 (96.1)	2 (0.5)	14 (3.5)
Every person treated with antibiotics is at an increased risk of antibiotic-resistant infection.	True	304 (74.0)	65 (15.8)	42 (10.2)
Antibiotic-resistant bacteria can spread from person to person.	True	184 (44.8)	163 (39.6)	64 (15.6)
Healthy people can carry antibiotic-resistant bacteria.	True	272 (66.2)	52 (12.7)	87 (21.1)
The use of antibiotics to stimulate growth in farm animals is legal in Oman.	False	51 (12.4)	129 (31.4)	231 (56.2)

*Represent the number of respondents (percentage) who answered as true, false, or unsure.

Supplemental Table S1 presents the median knowledge scores alongside the proportion of respondents achieving a full knowledge score across demographic categories. Overall median knowledge scores were significantly higher among respondents aged 46–55 years [6.5 (5.0–8.0), p=0.001] and Senior Specialist Pharmacists [7.0 (6.0–7.0), p<0.001]. When evaluating the proportion of participants achieving a full score (8/8), significant differences were observed by age and professional experience (p<0.001). Respondents aged 36–45 years (55&percnt;) and those with 16–20 years of practice (45&percnt;) comprised the highest proportion of full-score achievers.

Supplemental Table S2 presents the findings of the univariable linear regression analysis examining associations between demographic characteristics and knowledge scores. Variables demonstrating statistically significant associations in the univariable analysis were subsequently included in the multivariable regression model to identify independent predictors while accounting for potential confounding effects.

Table 3 outlines, the adjusted multivariable regression analyses. Male pharmacy professionals had significantly higher scores than female pharmacy professionals (β = 0.51, *p* = 0.001). While age groups showed differences in the univariable analysis, none remained significant after adjustment. Professional rank was positively associated with scores, with pharmacists (β = 0.48, *p* = 0.002) and senior specialist pharmacists (β = 1.20, *p* < 0.001) scoring higher than assistant pharmacists. Years of practice demonstrated a positive gradient, with significant differences observed among those with 11–15 years (β = 0.72, *p* = 0.006), 16–20 years (β = 0.97, *p* = 0.002), 21–25 years (β = 1.09, *p* = 0.007), and >25 years (β = 1.21, *p* = 0.037) of experience compared to those with 0–2 years.

**Table 3.** Multivariable linear regression analysis of the relationship between knowledge scores and demographic characteristics of respondents.

Variable		Unstandardised coefficient (β)	95% Confidence Interval (CI)	p-value
Gender	Female	–	(Reference)	
	Male	0.51	(0.21 to 0.82)	***0***.***001***
Age	24–35 years	–	(Reference)	
	36–45 years	−0.10	(−0.44 to 0.24)	0.561
	46–55 years	0.09	(−0.57 to 0.76)	0.789
Profession	Assistant Pharmacist	–	(Reference)	
	Pharmacist	0.48	(0.18 to 0.78)	***0***.***002***
	Senior Specialist Pharmacist	1.20	(0.71 to 1.69)	***<0***.***001***
Years of practice	0–2 years	–	(Reference)	
	3–5 years	0.31	(−0.24 to 0.85)	0.270
	6–10 years	0.47	(−0.03 to 0.97)	0.064
	11–15 years	0.72	(0.21 to 1.24)	***0***.***006***
	16–20 years	0.97	(0.37 to 1.57)	***0***.***002***
	21–25 years	1.09	(0.30 to 1.88)	***0***.***007***
	>25 years	1.21	(0.07 to 2.35)	***0***.***037***

Note: β represents the unstandardised linear regression coefficient, indicating the mean difference in overall knowledge score compared with the respective reference category (marked as Reference). Numbers in parentheses represent the 95% Confidence Intervals (CI). Statistical significance is defined as a two-tailed *p*-value less than 0.05.

Table 4 illustrates that nearly 91% of respondents reported either agreeing or strongly agreeing that they had a good understanding of the concept of antibiotic resistance. Above 91% of the respondents reported having sufficient information about antibiotic use and how to provide others with information about antibiotic resistance.

**Table 4.** Respondents’ perceived knowledge (n = 411).

	SA (%)	A (%)	D (%)	SD (%)	N/A (%)	U (%)	IDU (%)
**Perceived Knowledge**							
I know what antibiotic resistance is	155 (38)	217 (53)	7 (1.7)	9 (2.2)	1 (0.2)	9 (2.2)	13 (3.2)
I know what information to give to individuals about prudent use of antibiotics and antibiotic resistance	136 (33)	246 (60)	7 (1.7)	6 (1.5)	2 (0.5)	13 (3.2)	1 (0.2)
I have sufficient knowledge about how to use antibiotics appropriately for my current practice	132 (32)	243 (59)	9 (2.2)	7 (1.7)	2 (0.5)	18 (4.4)	0 (0)
**Opportunity**							
I have easy access to guidelines I need on managing infections	58 (14)	202 (49)	43 (10)	10 (2.4)	12 (2.9)	73 (18)	13 (3.2)
I have easy access to the materials I need to give advice on prudent antibiotic use and antibiotic resistance	51 (12)	246 (60)	37 (9.0)	13 (3.2)	10 (2.4)	53 (13)	1 (0.2)
I have good opportunities to provide advice on prudent antibiotic use to individuals	69 (17)	269 (65)	20 (4.9)	7 (1.7)	7 (1.7)	34 (8.3)	5 (1.2)
**Motivation to initiate antibiotic prescriptions**							
I know there is a connection between my dispensing of antibiotics and emergence and spread of antibiotic-resistant bacteria	106 (26)	225 (55)	20 (4.9)	10 (2.4)	5 (1.2)	30 (7.3)	15 (3.6)
I have a key role in helping control antibiotic resistance	111 (27)	195 (47)	12 (2.9)	15 (3.6)	16 (3.9)	56 (14)	6 (1.5)
**One Health: environmental and animal health factors that are important in contributing to antibiotic resistance in bacteria from humans**							
Environmental factors such as wastewater in the environment are important in contributing to antibiotic resistance in bacteria from humans	42 (10)	173 (42)	31 (7.5)	10 (2.4)	21 (5.1)	95 (23)	39 (9.5)
Excessive use of antibiotics in livestock and food production is important in contributing to antibiotic resistance in bacteria from humans	71 (17)	183 (45)	30 (7.3)	20 (4.9)	7 (1.7)	73 (18)	27 (6.6)

Abbreviations: SA: strongly agree; A: agree; D: disagree; SD: strongly disagree; N/A: not applicable; U: undecided; IDU: I do not understand.

#### 3.1.3. Opportunity and motivation

Over 63% of respondents agreed or strongly agreed that they had easy access to guidelines, and 72% agreed or strongly agreed that they could access materials to advise others on resistance. In addition, 82% reported having the opportunity to counsel individuals on prudent antibiotic use (Table 4). Table 4 shows that 81% of respondents agreed or strongly agreed they recognised the relationship between antibiotic dispensing and resistance spread, and 74% acknowledged their significant role in managing antibiotic resistance.

#### 3.1.4. One Health

Table 4 indicates that around 62% of respondents agreed or strongly agreed that the misuse of antibiotics in livestock and food production fosters resistance in human pathogens. Only 52% recognised environmental factors (e.g. wastewater) as contributors, 56% expressed uncertainty regarding the legality of antibiotic use for growth promotion in animal farming, and only 31.4% answered correctly (Table 2).

#### 3.1.5. Behaviour

Table 5 displays the behaviour of pharmacy professionals in promoting prudent antibiotic use. In accordance with national regulations in Oman, antibiotic dispensing across all healthcare facilities is strictly prescription-only. Out of the responses, 59.9% reported dispensing an antibiotic at least once daily in the past week. About 11.4% of participants provided resources on appropriate antibiotic use or infection management at least once and more than once a day in the past week. In comparison, 44.5% provided advice on prudent antibiotic use or infection management at least once a day in the past week.

**Table 5.** The frequency of pharmacy professionals dispensing antibiotic prescriptions and information regarding the prudent use of antibiotics along with thire antibiotic behaviour (n = 411).

Item	>QD (%)	>QW (%)	NVR (%)	R (%)	QD (%)	QW (%)	N/A (%)	IDR (%)
How often did you dispense antibiotics during the last one week?	232 (56.4)	60 (15)	26 (6.3)	17 (4.1)	14 (3.4)	7 (1.7)	32 (7.8)	23 (5.6)
How often did you give out resources (e.g. leaflets or pamphlets) on prudent antibiotic use or management of infections to individuals during the last one week?	38 (9.2)	24 (5.8)	93 (23)	139 (34)	9 (2.2)	32 (7.8)	31 (7.5)	45 (11)
How often did you give out advice related to prudent antibiotic use or management of infections to an individual during the last one week?	163 (40)	69 (17)	18 (4.4)	61 (15)	20 (4.9)	34 (8.3)	25 (6.1)	21 (5.1)

Abbreviations: QD: once a day; QW: once a week; NVR: never; R: rarely; N/A: not applicable; IDR: I do not remember.

According to Supplemental Table S3, 57.2% of pharmacy professionals reported being informed about how to avoid unnecessary antibiotic dispensing. Over 92.3% of them stated that knowledge helped them change their opinion about this, and roughly 95.9% claimed that knowledge altered how they dispensed antibiotics.

Regarding sources of information, 60% of participants received workplace guidance on avoiding unnecessary antibiotic prescriptions. Consequently, 51% of these individuals reported a shift in their perspectives. Furthermore, approximately 27% of participants gained knowledge from conferences, and only 21% reported changing their perspectives on avoiding unnecessary antibiotic use (Figure 1).

**Figure 1.**
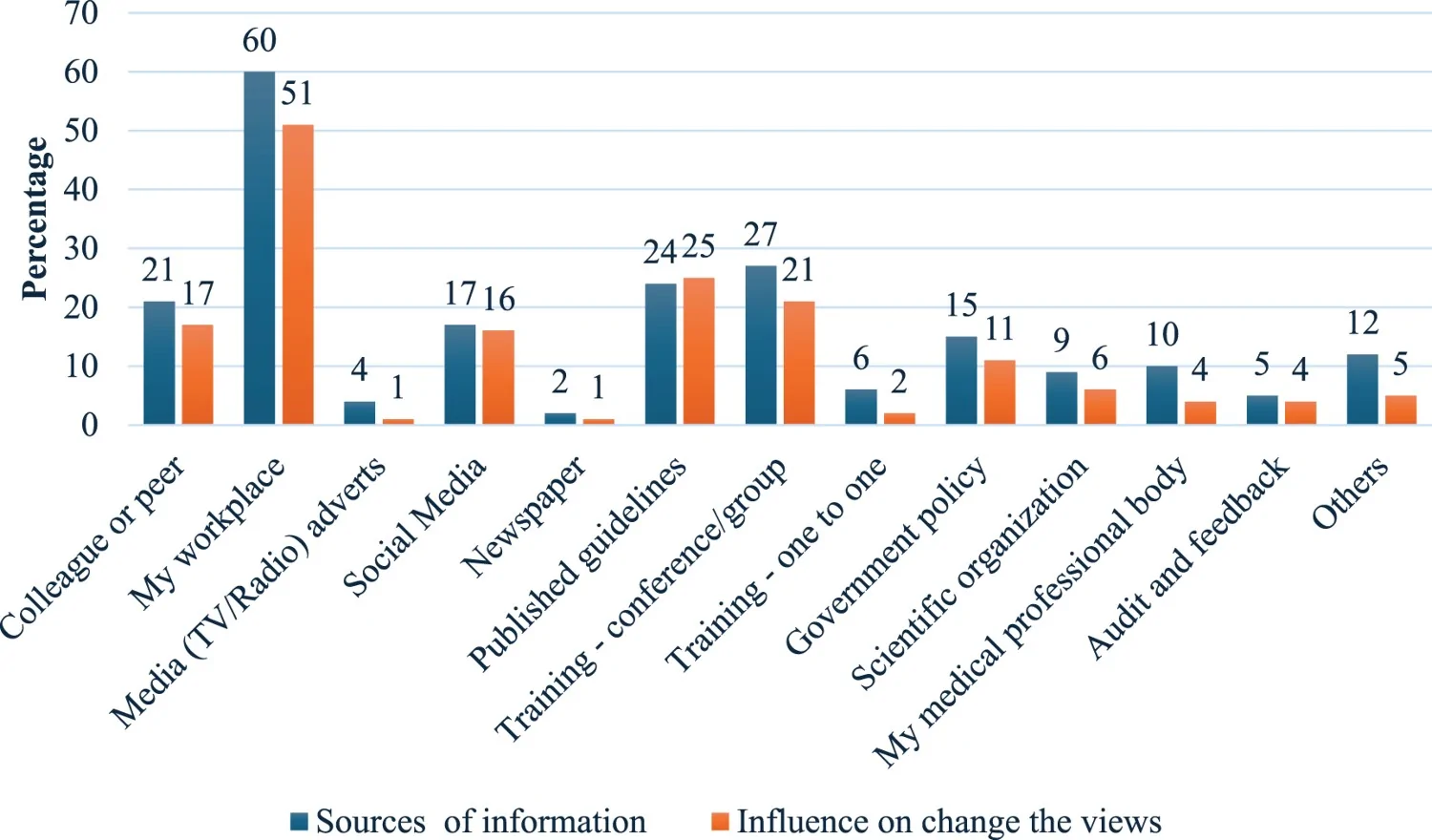
Sources of information about avoiding unnecessary dispensing of antibiotics and their influence on changing pharmacy professionals’ views (n = 411).

#### 3.1.6. Awareness of the Oman National Action Plan, national initiatives

Among the pharmacy professionals questioned, 264 (64%) were aware of the Oman National Action Plan on antibiotic resistance, 24 (6%) of them were unaware of it, and 123 (30%) were unsure whether such a plan existed in the country.

Awareness initiatives on antibiotics and resistance revealed that 40% of respondents were familiar with national or regional infection treatment guidelines. Another 38% reported learning about them through posters, leaflets, or events linked to antibiotic awareness programmes. Approximately 36% recognised World Antibiotic Awareness Week, while only 8% indicated no knowledge of such initiatives (Supplemental Table S4).

#### 3.1.7. Resources for managing infections and information gaps highlighted by the pharmacy professional

To control infections, about 44% of respondents used clinical practice guidelines, and 40% used publications and professional resources (Supplemental Table S5). Furthermore, only 27% of respondents reported depending on their previous clinical experience.

As shown in Supplemental Fig. S1, pharmacy professionals reported the following information needs: 58% wished to know more about antibiotic resistance, 54% wanted greater knowledge of which antibiotics are used for specific medical conditions, 37% expressed a need to enhance their understanding of appropriate antibiotic use, and 31% sought information on how to obtain prescribed antibiotics. Additionally, 40% indicated a need to understand how the health of people, animals, and the environment is interconnected.

## 4. Discussion

This study, conducted among pharmacy professionals employed in MOH institutions, found that their actual knowledge was moderate overall. The study revealed high levels of knowledge (>89%) among respondents concerning antibiotics’ ineffectiveness against viruses, the dangers of misuse, and their side effects. On the other hand, it showed poor knowledge in the spread of resistance (45%) and its use as growth promoters in farm animals (32%). It is similar to other study findings carried out in Jordan, which show high actual knowledge on antibiotic effectiveness and side effects (>87%), limited understanding of how resistance spreads (52.9%), and its legal use as growth promoters in farm animals (only 19%) (Al-Taani et al., 2022). These findings suggest that while healthcare professionals may possess adequate basic knowledge of antibiotic use, important gaps remain in the broader understanding of antimicrobial stewardship. Such deficiencies, along with limited access to training and institutional support, may limit effective participation in antimicrobial stewardship initiatives. Targeted educational programmes are therefore essential to strengthen knowledge and support implementation (Chetty & Leigh-de Rapper, 2023; Nasr et al., 2019; Saha et al., 2019).

### 4.1. Application of the COM-B behavioural framework

To systematically understand these findings and translate them into concrete policy, the data were evaluated using the COM-B behavioural framework, which explains behaviour as the interaction between capability, opportunity, and motivation (Michie et al., 2011). In addressing capability gaps, participants demonstrated strong psychological capability in basic antibiotic use, but significant gaps remain in resistance transmission pathways, environmental factors, and One Health principles. This deficit requires targeted, multi-disciplinary One Health and AMR training modules integrated into continuous professional development (CPD) frameworks and pharmacy university curricula across Oman.

When addressing opportunity gaps, although basic clinical guidelines are currently available, unequal regional and professional exposure highlights distinct institutional bottlenecks. Resolving these discrepancies demands enhanced workplace AMS support, localised clinical pathway access, and standardised digital tools distributed uniformly across all governorates to ensure equitable resource opportunity. Finally, in addressing motivation gaps, the data revealed a clear mismatch between high self-perceived confidence and lower objective knowledge, indicating potential professional overconfidence that could stall future learning. Overcoming this barrier requires the implementation of regular clinical audit and feedback loops, peer performance benchmarking, and formal professional incentives integrated within the Ministry of Health appraisal framework to maintain active, reflective motivation among pharmacy professionals.

### 4.2. Professional experience and contextual predictors within the Omani system

Our baseline knowledge findings closely mirror regional trends observed across neighbouring Gulf Cooperation Council (GCC) countries. Similar to recent findings in Saudi Arabia and Qatar, pharmacists consistently demonstrate high actual knowledge regarding basic pharmacology and patient counselling but possess limited structured training and variable awareness of systemic AMS application (Abdallah et al., 2025; Al Qamariat & Almaghaslah, 2021; Alshehri & Khawagi, 2025; Nasr et al., 2019).

Furthermore, our findings show that approximately 91% of pharmacy professionals reported awareness of antibiotic resistance. However, a significant discrepancy remains between self-perceived awareness and objectively assessed knowledge a trend documented internationally, where high professional self-confidence often masks gaps in complex microbiology or resistance mechanisms (Gajdacs et al., 2020) (Briquet et al., 2023). This mismatch could have important implications for Omani practice, as individuals who perceive themselves as sufficiently knowledgeable may be less inclined to pursue further training, highlighting the need for mandatory competency-based AMS evaluations rather than self-reported assessments (Gillani et al., 2022; Jarab et al., 2024; Ng et al., 2021).

The higher knowledge scores observed among senior pharmacists and those with longer professional experience reflect the structural organisation of the Omani healthcare system. In Oman, senior clinical and specialist pharmacists are routinely integrated into multidisciplinary hospital committees and participate directly in institutional stewardship protocols. This advanced professional exposure, combined with targeted MOH clinical pathways, naturally deepens their expertise over time compared to junior staff or assistant pharmacists, who may have fewer opportunities to engage in complex clinical interventions(Khumra et al., 2021; Teoh et al., 2020). Differences across practice settings and healthcare institutions may be associated with variability in stewardship implementation, institutional support, and access to AMR-related education and training opportunities (Jarab et al., 2024; Salsgiver et al., 2018).

### 4.3. One Health awareness and the National Action Plan

In our findings, knowledge of environmental and animal health factors was limited to 52% and 62% among pharmacy professionals, and only 31.4% correctly understood that the use of antibiotics for growth promotion in animal farming is illegal. This highlights the need to integrate One Health into pharmacy education to strengthen antimicrobial stewardship, support multidisciplinary roles, and enhance public health impact, despite challenges such as faculty training and curriculum redesign (Rajiah et al., 2025; Sjoberg et al., 2023). The limited awareness of One Health principles identified in this study is a significant concern for antimicrobial resistance control efforts. Effective AMR containment requires human, animal, and environmental health. Antibiotic misuse in agriculture and livestock, as well as environmental contamination, can contribute to the emergence and spread of resistant organisms (Berendonk et al., 2015). The relatively low level of awareness observed among pharmacy professionals may limit their ability to engage effectively in broader AMR prevention and public health initiatives, which require systems thinking to understand the multiscale dissemination of resistance (Arnold et al., 2024). Similar gaps in One Health understanding have been reported in other regional and international studies, where awareness of the formal One Health concept remains limited despite general recognition of its core principles (Wu et al., 2023). These findings highlight the need for greater integration of One Health concepts into healthcare education and antimicrobial stewardship training programmes (Czyz & Al-Mazroa Smith, 2023). Strengthening undergraduate curricula, continuous professional development activities, and interdisciplinary training initiatives is essential, as education plays a critical role in improving AMR awareness and stewardship practices (Castro-Sánchez et al., 2024; WHO, 2018). In Oman, the National Action Plan on Antimicrobial Resistance adopts a One Health approach and highlights awareness-raising, capacity building, and education as key strategic priorities to improve antimicrobial stewardship across the human, animal, and environmental health sectors (Ministry of Health & Ministry of Agriculture & Fisheries, 2016).

In our study, 72% of pharmacy professionals reported easy access to information, and 82% had good opportunities to provide advice on antibiotic resistance. While 40% reported giving advice to patients more than once daily. In Pakistan, 90.6% of community pharmacies were operating without an on-site qualified pharmacist, and non-qualified personnel demonstrated poor AMR knowledge, and patient pressure was common, with 74.4% requesting antibiotics without prescriptions (Waseem et al., 2019).

Our study shows that 81% of pharmacy professionals are aware of the link between dispensing and antibiotic resistance, and 74% agree that they play a key role in controlling it. Another study reported that while over 60% of pharmacists dispense antibiotics without a prescription, hospital pharmacists tend to maintain more positive beliefs regarding AMR compared to community pharmacists (Chan et al., 2022).

Under the One Health approach, controlling antibiotic resistance requires managing antibiotic use in both humans and animals (Khamis et al., 2023). Although national regulations in Oman ban the use of antibiotics as animal growth promoters (Ministry of Health & Ministry of Agriculture & Fisheries, 2016), only 31.4% of our respondents were aware of this rule. This low awareness highlights a clear gap in One Health education among pharmacy professionals. To support national stewardship goals, health and agricultural authorities should include basic veterinary regulations in pharmacy training and work together to ensure that animal antibiotics are dispensed only with a valid prescription (Ministry of Health & Ministry of Agriculture & Fisheries, 2016).

### 4.4. Clinical behaviour and regulatory strengths of the Omani healthcare sector

In terms of clinical behaviour, approximately 56% of pharmacy professionals dispensed antibiotics more than once per day, and 15% dispensed them more than once per week, placing them in a key position to implement antimicrobial stewardship interventions (Broom et al., 2015; Dickerson et al., 2000; Lambert et al., 2024). Crucially, because this sample is entirely drawn from the government healthcare sector, all reported dispensing practices are strictly prescription-bound in accordance with Oman's strict Ministry of Health regulations. Over-the-counter, non-prescription antibiotic sales are legally prohibited and tightly enforced across both public and private sectors in Oman (Alajmi et al., 2023). This high frequency places Omani pharmacy professionals in a powerful position to implement impactful AMS interventions, such as formal prescription reviews, targeted patient counselling, and dose optimisation. Although 63% of respondents in our study reported easy access to guidelines, this proportion remains lower than the approximately 82% guideline utilisation reported among pharmacists in South Africa, indicating possible gaps in accessibility, implementation, or awareness of therapeutic guidance in the Omani context (Balliram et al., 2021).

Our results show that the pharmacy professionals rarely (34%) or never (23%) distributed pamphlets to advise and educate the public. In contrast, about 40% of the counselling provided focused on antibiotics and infection management, with such advice delivered multiple times per day. This indicates that while pharmacists are actively engaged in verbal counselling, their contribution to distributing written educational materials remains limited, and greater involvement in pamphlet distribution could strengthen public education efforts. In a previous study, it was shown that the clinical pharmacist played a central role in evaluating the effectiveness of educational interventions, ensuring the accuracy of content, and monitoring changes in public awareness and antibiotic use behaviours. It demonstrated a significant improvement in awareness when video-based education was used, highlighting the potential of technology-driven approaches. Such evidence is directly relevant to our work, as it supports integrating innovative educational tools alongside traditional methods to enhance public engagement and reduce irrational antibiotic use (Radhakrishnan et al., 2023).

In our results, we found that pharmacy professionals who received education on avoiding unnecessary antibiotic dispensing changed their views and practices, with around 95% reporting changes in practice. In another study, pharmacists demonstrated good knowledge (70.6%) and positive perceptions (over 90%) of antibiotics and antimicrobial resistance, but poor dispensing practices (91.3%). As a consequence, the authors recommend strengthening regulations, continuous education, and monitoring to improve antimicrobial stewardship and reduce AMR risks (Akande-Sholabi et al., 2023). It has been recommended that law enforcement and penalties for illegal antibiotic sales be strengthened, improving public and pharmacist education on proper antibiotic use and AMR, expanding health insurance coverage, promoting family doctor systems and remote consultations to improve access to appropriate medical advice and reduce self-medication, and leveraging social media campaigns to reach low-education and freelance workers to increase awareness and encourage responsible antibiotic use (Nguyen et al., 2022).

Our findings showed that the pharmacy professionals’ first source of information is the workplace, followed by training/conference programmes, and then guidelines. Systematic reviews have shown that educational interventions targeting primary healthcare professionals are cost-effective, improving prescribing practices, reducing unnecessary antibiotic use, and enhancing patient outcomes. The findings support investing in guideline-based education, communication skills training, and multifaceted programmes, which not only lower costs but also promote rational antibiotic use. Policymakers and healthcare leaders should prioritise these strategies to combat AMR, particularly in low- and middle-income countries where misuse is prevalent (Rocha et al., 2022).

The study found low awareness of initiatives on antibiotic resistance in Oman. Respondents reported familiarity with national or regional guidelines for managing infections, and conferences and workshops focus on tracking AMR, and national posters or leaflets on antibiotic awareness. Oman has established antimicrobial stewardship (AMS) initiatives and ongoing national efforts to strengthen their implementation (Khamis et al., 2023; Nashwan et al., 2024). In Oman, Pharmacist-led clinical interventions have demonstrated significant success, with 97% of 2,032 analysed interventions accepted by treating physicians; nearly 30% of these accepted interventions specifically targeted antimicrobial prescribing (Al-Maqbali et al., 2022). Another study found that 26% of pharmacist interventions concerned antibiotics, with around 60% occurring in critical care units, likely reflecting the high intensity of antimicrobial use in these settings and the need for dose optimisation, avoidance of unnecessary antimicrobial use, and prevention of antimicrobial-related complications (Salman et al., 2021).

Our results show that pharmacy professionals most commonly used clinical guidelines (44%), followed by published research (40%) and, less frequently, their clinical experience (27%) to manage patient infections. In contrast, other studies report that continuing education is the primary source of information, followed by clinical guidelines (Al-Taani et al., 2022).

These findings provide direct, actionable insights to strengthen AMS policy and practice across Oman’s public healthcare system. At the policy level, the marked knowledge gap across professional categories underscores the need for tailored, competency-based AMS frameworks rather than uniform continuing education, particularly to train assistant pharmacists who comprise 41.1% of the workforce. Furthermore, limited awareness of the Oman National Action Plan and One Health principles highlights an urgent need for health authorities to mandate intersectoral AMR modules within university pharmacy curricula and professional re-licensure (Ministry of Health & Ministry of Agriculture & Fisheries, 2016). Policy should also drive the integration of clinical decision-support tools such as mandatory indication entry and automated timeout alerts – directly into the national *Al Shifa* electronic health system (Khamis et al., 2023).

In clinical practice, on-site interventions should be prioritised. Because workplace guidance was the primary information source and led nearly 96% of recipients to modify their dispensing practices, healthcare facilities should establish routine audit-and-feedback rounds and case-based discussions (Salman et al., 2021). These objective assessments are essential to bridge the observed gap between high self-perceived confidence and actual knowledge deficits. Finally, while 44.5% of respondents counsel patients daily, distribution of written educational materials remains low. Supplying outpatient dispensing counters with standardised bilingual leaflets or digital QR-code resources would effectively reinforce verbal counselling and reduce patient expectations for unnecessary antibiotics.

AMS programme in Oman have demonstrated measurable success in major tertiary hospitals. Multidisciplinary initiatives at The Royal Hospital significantly reduced methicillin-resistant *Staphylococcus aureus* (MRSA) and carbapenem-resistant pathogens, generating sustained annual savings of $70,000 to $80,000 USD (Khamis et al., 2023). Similarly, prospective audit-and-feedback at Sultan Qaboos University Hospital (SQUH) lowered multidrug-resistant organism rates and achieved nearly $200,000 USD in annual net cost savings from antibiotic interventions alone (Helmi et al., 2024; Salman et al., 2021). In specialised pediatric care, a targeted programme at Khoula Hospital reduced neonatal antibiotic days of therapy by 64.8% through 48-hour timeouts and restricted broad-spectrum prescribing (Malviya et al., 2024).

Despite these hospital-level successes, nationwide implementation faces clear operational hurdles. National evaluations have highlighted marked regional disparities, noting that peripheral facilities often lack dedicated stewardship teams, specialised clinical pharmacists, and electronic decision support (Khamis et al., 2023; Ministry of Health & Ministry of Agriculture & Fisheries, 2016). These systemic challenges are reinforced by (Alqayoudhi et al., 2026), who documented wide prescribing variations across Omani neonatal intensive care units driven by individual hospital habits rather than uniform protocols. This lack of standard practice explains why 54% of our survey respondents requested clearer, indication-specific guidance. Moreover, documented guideline adherence gaps among Oman Medical Speciality Board pediatric residents (Alshukaili et al., 2025) highlight the necessity of ward-based clinical oversight, validating our finding that Senior Specialist Pharmacists (β=1.20,p<0.001) serve as vital clinical safeguards during medical rounds.

Finally, stewardship initiatives in Oman remain mainly concentrated in inpatient tertiary centres, leaving ambulatory and primary care under-supported (Al Hoqani et al., 2026). This gap is increasingly critical given the rise in outpatient resistant pathogens, such as ESBL-producing *Escherichia coli* in uncomplicated urinary tract infections, which limits oral treatment choices and increases hospital referrals (Al-Mufarji et al., 2026). Because 44.5% of our respondents provide verbal counselling daily while over 57% lack written patient education materials, national AMS strategies must expand beyond inpatient restrictions to equip outpatient pharmacy professionals with practical educational tools and point-of-care decision support.

### 4.5. Strengths and limitations

This study has several important strengths. To our knowledge, it is one of the few studies to examine pharmacy professionals’ knowledge, opinions, and practices regarding antimicrobial resistance and antimicrobial stewardship in Oman. The survey was sent to all eligible pharmacy professionals across different settings, thereby making the sample more diverse and representative. Also, the number of participants exceeded the minimum required, which makes the results more reliable. However, several methodological limitations must be explicitly acknowledged. First, the study is subject to potential self-selection bias, as individuals with a baseline interest or higher existing competence in antimicrobial stewardship may have been more inclined to complete the survey. Second, because the data relied entirely on self-reported behaviour rather than objective dispensing data or direct prescription audits, the true clinical practices could not be independently verified. This reliance on self-reporting introduces the possibility of social desirability bias, in which participants may report behaviours that align with idealised guidelines rather than their daily routine. Third, despite exceeding the calculated power requirements, the relatively low response rate observed across certain institutions could limit the generalizability of the findings beyond Ministry of Health (MOH) pharmacy professionals to private-sector practitioners. Furthermore, because the cross-sectional study design collected data at only a single point in time, it is not possible to establish definite temporal cause-and-effect relationships. Finally, while the adapted questionnaire underwent bilingual expert validation and preliminary pilot testing, formal back-translation and cognitive interviewing were not performed. Despite these limitations, this study provides critical, high-utility baseline information that can directly support future antimicrobial stewardship programmes and health policy research in Oman.

## 5. Conclusion

This study found that pharmacy professionals in Oman generally demonstrated good knowledge and positive perceptions regarding antibiotic use and AMR, with most reporting access to guidelines and opportunities to provide patient counselling. However, important gaps remained in understanding resistance spread, One Health concepts, and national AMR initiatives; although respondents who received relevant information frequently reported changes in their dispensing practices, the appropriateness of those practices was not directly assessed. These findings underscore the important role of pharmacists in antimicrobial stewardship and the need to strengthen the translation of knowledge into clinical practice. Future interventions should prioritise integrating AMR and One Health principles into undergraduate pharmacy curricula, alongside strengthening continuous professional development programmes focused on antimicrobial stewardship. In addition, increasing institutional awareness of national AMR strategies and enhancing structured training on guideline-based prescribing support are essential to improve engagement in stewardship activities. Strengthening these targeted educational and policy-driven interventions may enhance appropriate antibiotic use, improve patient safety, and contribute to national and regional efforts to combat antimicrobial resistance.

## Supplementary Material

Supplemental Material

## Data Availability

The data supporting the findings of this study are available from the corresponding author upon reasonable request, subject to relevant research approvals in Oman.

## Al declaration

The authors used Google Gemini 3.5 to improve readability, grammar, and syntactic structure during manuscript preparation. All suggested modifications were reviewed by the authors, who take full accountability for the published text.
