# Supplementary material for: Pharmacy professionals’ knowledge, attitudes, and behaviours regarding antibiotic use and antimicrobial resistance in Oman
Source: J Pharm Policy Pract. 2026 Sep 30;19(1):2737505. doi: 10.1080/20523211.2026.2737505 (PMC13629792; doi:10.1080/20523211.2026.2737505)
Supplement: Supplementary file 1 [file JPPP_A_2737505_SM3569.docx]

**Supplementary**

**Table S1. Predictors of actual knowledge scores among pharmacy** **professionals (n=411).**

| **Variable** | **Score (Range 0–8)** | **No. of Respondents** | **p-value** | **No, N = 369^1^** | **Yes, N = 42^1^** | **p-value^2^** |
| --- | --- | --- | --- | --- | --- | --- |
| **Gender** |  |  |  |  |  |  |
| Female | 5.0 (5.0 to 6.0) | 302 (73.5) |  | 285 (77%) | 17 (40%) |  |
| Male | 6.0 (5.0 to 7.0) | 109 (26.5) | ***<0.001*** | 84 (23%) | 25 (60%) | ***<0.001*** |
| **Age** |  |  |  |  |  |  |
| 24-35 years | 6.0 (4.5 to 6.0) | 203 (49.4) |  | 195 (53%) | 8 (19%) |  |
| 36-45 years | 6.0 (5.0 to 7.0) | 178 (43.3) | ***0.001*** | 155 (42%) | 23 (55%) | <***0.001*** |
| 46-55 years | 6.5 (5.0 to 8.0) | 30 (7.3) |  | 19 (5.1%) | 11 (26%) |  |
| **Governorate** |  |  |  |  |  |  |
| Ad Dakhiliyah | 6.0 (5.0 to 7.0) | 32 (7.8) |  | 29 (7.9%) | 3 (7.1%) | 0.336 |
| Ad Dhahirah | 6.0 (5.0 to 7.0) | 39 (9.5) |  | 35 (9.5%) | 4 (9.5%) |  |
| Al Batinah North | 5.5 (5.0 to 7.0) | 12 (2.9) |  | 11 (3.0%) | 1 (2.4%) |  |
| Al Batinah South | 6.0 (5.5 to 7.0) | 15 (3.6) |  | 14 (3.8%) | 1 (2.4%) |  |
| Al Buraymi | 5.0 (4.0 to 6.0) | 6 (1.5) |  | 6 (1.6%) | 0 (0%) |  |
| Al Wusta | 6.5 (6.0 to 7.2) | 20 (4.9) | ***0.005*** | 15 (4.1%) | 5 (12%) |  |
| Ash Sharqiyah North | 6.0 (5.0 to 7.0) | 41 (10.0) |  | 36 (9.8%) | 5 (12%) |  |
| Ash Sharqiyah South | 5.0 (5.0 to 6.0) | 30 (7.3) |  | 28 (7.6%) | 2 (4.8%) |  |
| Dhofar | 6.0 (6.0 to 7.0) | 20 (4.9) |  | 16 (4.3%) | 4 (9.5%) |  |
| Musandam | 6.0 (6.0 to 6.5) | 8 (1.9) |  | 6 (1.6%) | 2 (4.8%) |  |
| Muscat | 5.0 (4.0 to 7.0) | 188 (45.7) |  | 173 (47%) | 15 (36%) |  |
| **Profession** |  |  |  |  |  | 0.052 |
| Assistant Pharmacist | 5.0 (5.0 to 6.0) | 169 (41.1) |  | 155 (42%) | 14 (33%) |  |
| Pharmacist | 6.0 (5.0 to 7.0) | 201 (48.9) |  | 182 (49%) | 19 (45%) |  |
| Senior Specialist Pharmacist | 7.0 (6.0 to 7.0) | 41 (10.0) | ***<0.001*** | 32 (8.7%) | 9 (21%) |  |
| **Place of practice** |  |  |  |  |  | 0.2 |
| Hospital (any hospital type) | 6.0 (5.0 to 7.0) | 231 (56.2) | 0.650 | 203 (55%) | 28 (67%) |  |
| Primary health centre | 6.0 (5.0 to 7.0) | 146 (35.5) |  | 133 (36%) | 13 (31%) |  |
| Secondary care centre (polyclinic or dialysis centre ) | 5.5 (5.0 to 6.0) | 34 (8.3) |  | 33 (8.9%) | 1 (2.4%) |  |
| **Years of practice** |  |  |  |  |  |  |
| 0-2 years | 5.0 (4.0 to 6.0) | 38 (9.2) |  | 38 (10%) | 0 (0%) |  |
| 3-5 years | 5.0 (4.0 to 6.0) | 55 (13.4) |  | 53 (14%) | 2 (4.8%) |  |
| 6-10 years | 5.0 (4.0 to 6.5) | 99 (24.1) |  | 91 (25%) | 8 (19%) |  |
| 11-15 years | 6.0 (5.0 to 7.0) | 112 (27.3) |  | 106 (29%) | 6 (14%) |  |
| 16-20 years | 6.0 (5.0 to 7.5) | 75 (18.2) | ***<0.001*** | 56 (15%) | 19 (45%) | ***<0.001*** |
| 21-25 years | 6.0 (5.0 to 7.0) | 24 (5.8) |  | 19 (5.1%) | 5 (12%) |  |
| >25 years | 6.5 (5.0 to 7.2) | 8 (1.9) |  | 6 (1.6%) | 2 (4.8%) |  |
| ^1^n (%) | | | | | | |
| Yes, indicates a full score | | | | | | |
| ^2^Pearson's Chi-squared test; Fisher's exact test | | | | | | |

**Table S2. Univariable linear regression** **analysis of the relationship between knowledge scores and demographic characteristics of respondents.**

| Variable |  | Unstandardized Coefficient ($\boldsymbol{\beta)}$ | 95% Confidence Interval (CI) | $\boldsymbol{p}$-value |
| --- | --- | --- | --- | --- |
| Gender | Female | - | (Reference) |  |
|  | Male | 0.75 | 0.45 to 1.05 | ***p<0.001*** |
| Age | 24-35 years | - | (Reference) |  |
|  | 36-45 years | 0.39 | 0.11 to 0.68 | ***p=0.006*** |
|  | 46-55 years | 0.89 | 0.35 to 1.43 | ***p=0.001*** |
| Governorate | Ad Dakhiliyah | - | (Reference) |  |
|  | Ad Dhahirah | -0.08 | -0.74 to 0.57 | p=0.802 |
|  | Al Batinah North | 0.17 | -0.76 to 1.09 | p=0.724 |
|  | Al Batinah South | 0.12 | -0.74 to 0.97 | p=0.789 |
|  | Al Buraymi | -0.75 | -1.97 to 0.47 | p=0.227 |
|  | Al Wusta | 0.70 | -0.08 to 1.48 | p=0.078 |
|  | Ash Sharqiyah North | 0.32 | -0.32 to 0.97 | p=0.326 |
|  | Ash Sharqiyah South | -0.35 | -1.05 to 0.35 | p=0.323 |
|  | Dhofar | 0.60 | -0.18 to 1.38 | p=0.131 |
|  | Musandam | 0.63 | -0.46 to 1.71 | p=0.257 |
|  | Muscat | -0.31 | -0.84 to 0.21 | p=0.239 |
| Profession | Assistant Pharmacist | - | (Reference) |  |
|  | Pharmacist | 0.14 | -0.15 to 0.42 | p=0.347 |
|  | Senior Specialist Pharmacist | 0.97 | 0.50 to 1.45 | ***p<0.001*** |
| Place of practice | Hospital (any hospital type) | - | (Reference) |  |
|  | Primary health centre | -0.02 | -0.31 to 0.28 | p=0.913 |
|  | Secondary care centre (polyclinic or dialysis centre) | -0.20 | -0.71 to 0.31 | p=0.441 |
| Years of practice | 0-2 years | - | (Reference) |  |
|  | 3-5 years | 0.25 | -0.32 to 0.82 | p=0.397 |
|  | 6-10 years | 0.46 | -0.06 to 0.98 | p=0.082 |
|  | 11-15 years | 0.77 | 0.26 to 1.28 | ***p=0.003*** |
|  | 16-20 years | 1.16 | 0.62 to 1.70 | ***p<0.001*** |
|  | 21-25 years | 1.10 | 0.39 to 1.80 | ***p=0.002*** |
|  | >25 years | 1.22 | 0.17 to 2.28 | ***p=0.023*** |

**Table S3.** **Number of respondents (%) who received information on avoiding unnecessary dispensing of antibiotics and, of those, the number (%) reporting that the information contributed to changing their views or practice.**

| Questions | Yes | No | Unsure |
| --- | --- | --- | --- |
|  | ***n* (%)** | ***n* (%)** | ***n* (%)** |
| In the last 12 months, do you remember receiving any information about avoiding unnecessary dispensing of antibiotics? (n=411) | 235 (57.2) | 60 (14.6) | 116 (28.2) |
| Did the information contribute to changing your views about avoiding unnecessary dispensing of antibiotics? (n=235) | 217 (92.3) | 10 (4.3) | 8 (3.4) |
| On the basis of the information you received, have you changed your practice on dispensing of antibiotics? (n=217) | 208 (95.8) | 6 (2.8) | 3 (1.4) |

**Table S4. Awareness of initiatives on antibiotic awareness and resistance (n=411).**

| Items | n (%) |
| --- | --- |
| TV or Radio advertising for the public | 76 (19) |
| Toolkits and resources for healthcare workers | 137 (33) |
| National or regional guidelines on management of infections | 163 (40) |
| Awareness raising from professional organizations | 128 (31) |
| Conference/Events focused on tackling antibiotic resistance | 156 (38) |
| National or regional posters or leaflets on antibiotic awareness | 159 (39) |
| Newspaper (national) articles on antibiotic resistance | 35 (9) |
| World Antibiotic Awareness Week | 149 (36) |
| I am not aware of any initiatives | 31 (8) |

| In the management of infections, which of these do you use regularly? | n (%) |
| --- | --- |
| Clinical practice guidelines | 181 (44) |
| Documentation from the pharmaceutical industry | 59 (14) |
| Medical representatives from industry | 19 (5) |
| Previous clinical experience | 110 (27) |
| Continuing education training courses | 99 (24) |
| Infection specialists | 82 (20) |
| Scientific journals | 90 (22) |
| Professional resources/publications | 163 (40) |
| Social media | 84 (20) |
| None of the above | 23 (6) |
| I do not know | 27 (7) |
| Others | 21 (5) |

**Table S5. Resources used for the management of patients with infections.**

**Figure S1**. **Topics that pharmacy professionals would like to receive more information on.**

|  | Percentage |
| --- | --- |
| Resistance to antibiotics | 58 |
| How to use antibiotics | 37 |
| Medical conditions for which antibiotics are used | 54 |
| Prescription of antibiotics | 31 |
| Links between the health of humans, animals and the environment | 40 |
| None | 1 |
| Others | 4 |
